# Emergence of new resistances to *Cydia pomonella Granulovirus*: insights from 12 years of monitoring

**DOI:** 10.3389/fphys.2026.1847124

**Published:** 2026-07-08

**Authors:** Myriam Siegwart, Sandrine Maugin, Léa Gingueneau, Samantha Besse

**Affiliations:** 1 INRAE, UR Plantes et Système de cultures Horticoles (PSH), Domaine Saint-Paul, Avignon, France; 2 Laboratoires Goëmar – site Natural Plant Protection (NPP), UPL group, Pau, France

**Keywords:** apple tree, baculovirus, bioassays, cartography, cross-resistance, codling moth

## Abstract

**Introduction:**

Codling moth resistance to the granulosis virus CpGV, a widely adopted biological control agent in apple orchards poses a significant challenge for growers. Since the first report of Type I resistance to the CpGV-M in Europe (2005), a monitoring system was set up in France to track resistance evolution and anticipate emergence to new viral variants.

**Methods:**

This study presents the results of 12 years of bioassay based monitoring of resistance to three commercially available CpGV isolates (CpGV-M, CpGV-V15 and CpGV-R5), representative of the virus genetic diversity.

**Results:**

Among 193 wild codling moth populations tested, 62.3% carried a significant number of individuals resistant to CpGV-M, 37.0% to CpGV-V15 and 13.3% to CpGV-R5. Despite mitigation recommendations implemented since the first detection of type I resistance, the prevalence of CpGV-M resistance has not declined over time. Similarly, resistance to CpGV-M + CpGV-V15 (Type IV) and CpGV-M + CpGV-V15 + CpGV-R5 (Type V) seems to be fairly stable in frequency from one year to the next. Populations carrying type I resistance have been detected in all French production basins and in northern Italy, while type IV and V resistances are localized to northwestern France and northern Italy.

**Discussion:**

The emergence of these resistances types underscore the urgency of revising deployment strategies for new granulovirus-based products. Effective solutions will require elucidating the underlying resistance mechanisms to inform countermeasures.

## Highlight

- Codling moths have developed multiple resistance to three CpGV isolates marketed in France.
- 62.3% of tested populations carried resistant individuals to CpGV-M, 37.0% to CpGV-V15 and 13.3% to CpGV-R5.
- Type I resistance is present in all the production basins monitored, while types IV and V are not.
- Resistance management must take into account mixtures of viral genotypes in commercial products.

## Introduction

Pest resistance to pesticides is a rapid micro-evolutionary phenomenon that arises when large populations of individuals are subjected to intense selection pressures (Hemingway et al., 2002). This phenomenon has been extensively studied for chemical insecticides, due to its significant impact on modern agriculture. While resistance to biocontrol methods is beginning to be documented, it remains less well understood because of the uniqueness of each of these adaptations (Siegwart et al., 2015). The most effective biocontrol methods are also the most widely used. In these circumstances, effective biocontrol products induce sufficient selection pressure on pests to reveal resistance. For instance, resistance to the bacterial *Bacillus thuringiensis* (Bt) toxins is largely described and developed various resistance mechanisms (Pardo-Lopez et al., 2013). It had been documented in numerous lepidopteran species, with laboratory-selected populations demonstrating a wide array of adaptive mechanisms. These mechanisms include altered protease activation of protoxins, toxin sequestration by glycolipids or esterases, elevated immune responses (such as increased melanization), and, most commonly, reduced toxin binding to midgut membranes due to mutations in receptor genes (e.g., cadherin, APN, ALP) or in the ABCC2 transporter (Pardo-Lopez et al., 2013).

The codling moth (*Cydia pomonella*) is one of the most important lepidopteran pests in apple orchards worldwide. The larvae of this lepidoptera feed on the fruit, rendering them unmarketable as fresh produce and creating entry points for pathogens, which also limits their potential for commercialization as processed products (Balachowsky, 1966). Infestations by codling moths can cause serious yield loss (Barnes, 1991), hence the extensive use of insecticides to control the population sizes of this pest. Its biology and exceptional adaptive capabilities, combined with the intense selection pressure exerted by the widespread and repeated use of insecticides, have enabled it to develop resistance to various insecticides, both synthetic and biological (Ju et al., 2021). The resistance mechanisms developed by this insect are diverse. They involve detoxification, modifications of molecular targets (Ju et al., 2021), and alterations in immune pathways (Olivares et al., 2023).

The Cydia pomonella Granulovirus (CpGV) was first isolated from codling moth larvae in Mexico in 1963, maned CpGV-M (Tanada, 1964). Extensive studies have demonstrated its effectiveness as a specific biocontrol agent, ensuring minimal impact on the environment and human health (Lacey and Thomson, 2004). Since the discovery of CpGV-M, prospection has expanded globally, leading to the identification of dozens of additional isolates with varying levels of virulence. These isolates are currently classified into 7 genotypic groups (from A to G), with CpGV-M belonging to group A (Fan, 2019). CpGV-M was marketed as a bioinsecticide in Europe since 1989, it was rapidly adopted by farmers. Following 20 years of intensive use of the Mexican isolate of the Cydia pomonella granulovirus (CpGV-M) (*Betabaculovirus cypomonellae*) as biocontrol agent in orchards, cases of resistance—referred to as “Type I” resistance—were detected in France and Germany in 2005 (Asser-Kaiser et al., 2007; Sauphanor et al., 2006). This resistance, characterized by a high resistance ratio (RR = 13,000) and a low fitness cost that was undetectable under laboratory conditions, quickly spread across Europe and was more recently reported in the United States (Fan et al., 2022). This resistance confers immunity against CpGV strains of genotypic group A, but can be overcome by strains containing genotypic group B to D (Gebhardt et al., 2014)(Tab. 1). This resistance is a major dominant resistance linked to the sexual Z chromosome (Asser-Kaiser et al., 2007; Berling et al., 2013; Olivares et al., 2023). In resistant individuals, despite their capacity to enter into the body of the larvae, the virus is not able to replicate and continue its life cycle. The properties of this resistance pose significant agronomic challenges, particularly in organic farming, where few effective alternatives against *C. pomonella* are available. This crisis prompted rapid innovation, leading to the commercialization of new CpGV-based products effective against CpGV-M resistant insects just six years after the first detections. The two viral isolates incorporated into these products are CpGV-R5 and CpGV-V15 (Gueli Alletti et al., 2017; Graillot et al., 2014), respectively composed by genotypic group A and E; and genotypic groups A, B and E (Tab. 1).

Two additional resistance types, termed Type II and Type III, were described in 2017, but in Europe they have not been detected beyond Germany (Sauer et al., 2017). These two types of resistance affect both CpGV-S and CpGV-M, but with a different inheritance pattern, the Type II is autosomal and dominant and type III show a mixture of a Z-linked and autosomal inheritance pattern (Sauer et al., 2017) The CpGV-S and CpGV-R5 isolates are relatively closely related, as they mostly belong to the same genotypic group (E).

The first studies on the CpGV resistance were conducted following the reporting of local cases of failure in codling moth control in Germany and France (Fritsch et al., 2005; Sauphanor et al., 2006). Then, a monitoring program were launched to establish a state of the play of Type I resistance in Europe (Zichová et al., 2013). The following cases of resistance (Type II and III) were also detected because of cases of failure in codling moth control despite the introduction of new isolates (Jehle et al., 2026). Monitoring resistance across time and space is essential for managing resistance and prolonging the effectiveness of products by implementing appropriate strategies. A study has shown that resistance monitoring yields give better results when conducted through collaboration between public and private stakeholders (R4P, 2021).

This study presents the results of 12 years of monitoring *C. pomonella* resistance to three CpGV main viral isolates currently available on the European market. The objective was both to detect emerging resistances at an early stage and to track spatial and temporal changes in resistance to CpGV-M. To achieve this, field populations were collected from orchards and exposed to the different viral isolates to evaluate their susceptibility. The commercial products currently in use are based on genotypic groups A, B, and E. CpGV-M, which belongs exclusively to genotypic group A, was used to monitor the evolution of Type I resistance. CpGV-R5 was selected to represent group E, as products such as Carpovirusine Evo2^®^ and Madex Max^®^ predominantly containing this genotypic group. CpGV-V15, a mixture of several genotypic groups, was included to assess the ability of codling moth populations to develop multiple resistance, particularly to group B (**Table 1**).

**Table 1:** List of CpGV isolates used in agriculture against the codling moth (*Cydia pomonella*).

Isolate	Some commercial products names	Genotypic group^a^	Commercialization (first date)^b^
CpGV-M	Madex, Carpovirusine, Carpovirusine Max, Granupom, Carpostop, Carpo 600, Cyd-X	A (100%)	World (1989)
CpGV-V22	Madex Twin, Madex HP		
CpGV-V01	Cyd-X HP, Madex Plus		
CpGV-V03	Madex Max	A (32%) + E^*^ (68%)	Europe (2024)
CpGV-R5	Carpovirusine Evo 2, Carpovirusine Ultra	A (33%) + E^*^ (67%)	World (2012)
CpGV-V14	Madex Primo	A (27%) + B (81%)	Europe (not yet)
CpGV-V15	Madex Top, Madex Pro	A (7%) + B (42%) + E^*^ (49%)	Europe (2013)
CpGV-V45	Madex Duo, Madex XLV	A (61%) + B (2%) + E^*^ (19%) + other (11%)	Europe, USA (2025)
CpGV-S	Virosoft CP4	E^*^ (100%)	USA, Canada (2000)

^a^ Median percentage of SNP variants specific to each genotypic group. The presence of recombinant viruses and as-yet undescribed groups explains why the sum of these values does not reach 100% (Fan, 2019). ^b^not all countries of the geographic area are concerned ^*^ The percentage of genotypic group E was inferred based on the data from Fan (2019) .

## Materials and methods

### Insects

#### Wild populations

We consider an insect population as the group of individuals trapped within the same orchard plot. In this study, we were able to conduct tests on 200 wild populations with the following distribution: 2013(11), 2014(17), 2015(3), 2016(13), 2017(8), 2018(20), 2019(1), 2020(34), 2021(8), 2022(18), 2023(35), 2024(32). Information on treatments, agricultural practices and GPS coordinates for these plots was obtained through surveys conducted with farmers. The majority of the sampled populations come from France (164), but a few (35) were also sampled in other European countries: Germany(2), Spain(3), Greece(1), Hungary(2), Italy(25), Czech Republic(1) and UK (2). The same populations were not necessarily sampled from one year to the next (Sup mat 1).

Wild populations were obtained by trapping individuals from commercial orchards using cardboard trap bands placed around apple tree trunks in July. The trap bands were collected in October and examined to extract only codling moth larvae. The larvae were then reconditioned in new cardboard strips and placed at 11 °C for 24 hours to allow them to rebuild their cocoon. Subsequently, they were stored at 4 °C for four months until the breaking of the diapause.

Diapausing larvae were then transferred to 23 °C under long-day photoperiod conditions (16 h light: 8 h dark) to induce pupation. Adults emerged after approximately ten days. Upon emergence, adults were sexed and placed in empty 1.5 L plastic water bottles, which had been cut at one end and covered with insect-proof mesh, at a density of 10 pairs per bottle.

**Figure 1:**
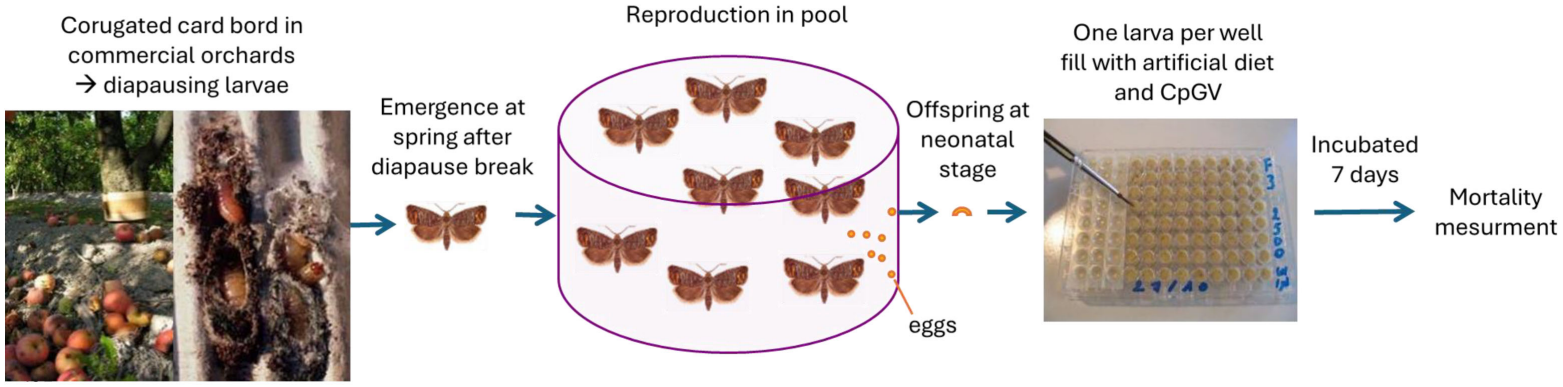
Diagram of the experimental setup for bioassays.

Inside each bottle, moist cotton pads were provided, and humidity was maintained by spraying water twice daily. After seven days, adults were transferred to new bottles, while the old ones were cut to collect the eggs laid directly on their inner walls. The pieces of cut bottles were then placed in containers with a moist cotton pad until the first neonate larvae hatched. Neonate larvae less than 12 hours old were used for bioassays (**Figure 1**).

#### Reference strain

The susceptible laboratory strain (Sv) originates from a population collected in “Les Vignères,” located in the south of France, and has been mass-reared on artificial diet (Guennelon et al., 1981) under laboratory conditions, with no exposure to insecticides for over 25 years.

#### Viruses

Three CpGV isolates commercially available in France were used for this monitoring: CpGV-M, CpGV-V15, and CpGV-R5. These isolates are representative of the composition of the commercial products Carpovirusine 2000™ (UPL), Madex Pro^®^ (Andermatt), and Carpovirusine Evo2™ (UPL), respectively. All viruses were stored at -20 °C and thawed immediately before use. Viral solutions were aliquoted to prevent repeated freezing and thawing cycles.

#### Bioassays

Microplate bioassays were performed to assess the susceptibility of various wild populations to three CpGV isolates commercialized in France for *Cydia pomonella* control.

The bioassay was conducted using 96-well plates filled with an artificial diet (Stonefly Heliothis^®^, Ward’s Science, USA). Each well received 6 µL of a viral solution at the discriminating dose or 6 µL of water for the control. A single neonate larva was carefully placed in each well using a fine brush, wells were sealed with Parafilm and the plates were incubated for seven days at 23 °C under a 16:8 h light:dark photoperiod. Larval mortality was recorded after seven days.

The susceptible laboratory strain (Sv) was included as a reference. A minimum of 20 larvae per isolate and per control condition were tested. The discriminating dose was determined annually by establishing a dose-mortality curve on the susceptible strain, corresponding to the lowest dose that kills all susceptible individuals. This dose remained unchanged over the 12 years of testing: 6250 GV/µL for CpGV-M, 6250 GV/µL for CpGV-R5, and 31250 GV/µL for CpGV-V15.

### Statistical analyses

Wild populations are categorized as resistant based on a Chi-square test comparing their corrected mortality with that of the susceptible reference strain Sv (thresholds of p<0.01 in red, 0.01<p<0.05 in orange, p>0.05 in green on the figures). The corrected mortality is calculated using Abbott’s formula. To compare corrected mortalities based on agricultural practices, an ANOVA was performed followed by a Tukey test. Statistical analyses as well as the maps were performed using RStudio 2023.12.1 and R software version 4.3.2 (R Core, 2013; RStudio, 2021). To determine whether there was a correlation between resistance to the 3 isolates, population are considered was resistant if the Chi-square test comparing it to the reference population was highly significant (p<0.01). Contingency table was created counting the cases of cross-resistance 2 by 2 (for example CpGV-M and CpGV-R5) in order to be able to carry out a new Chi-square test.

## Results

**Figure 2:**
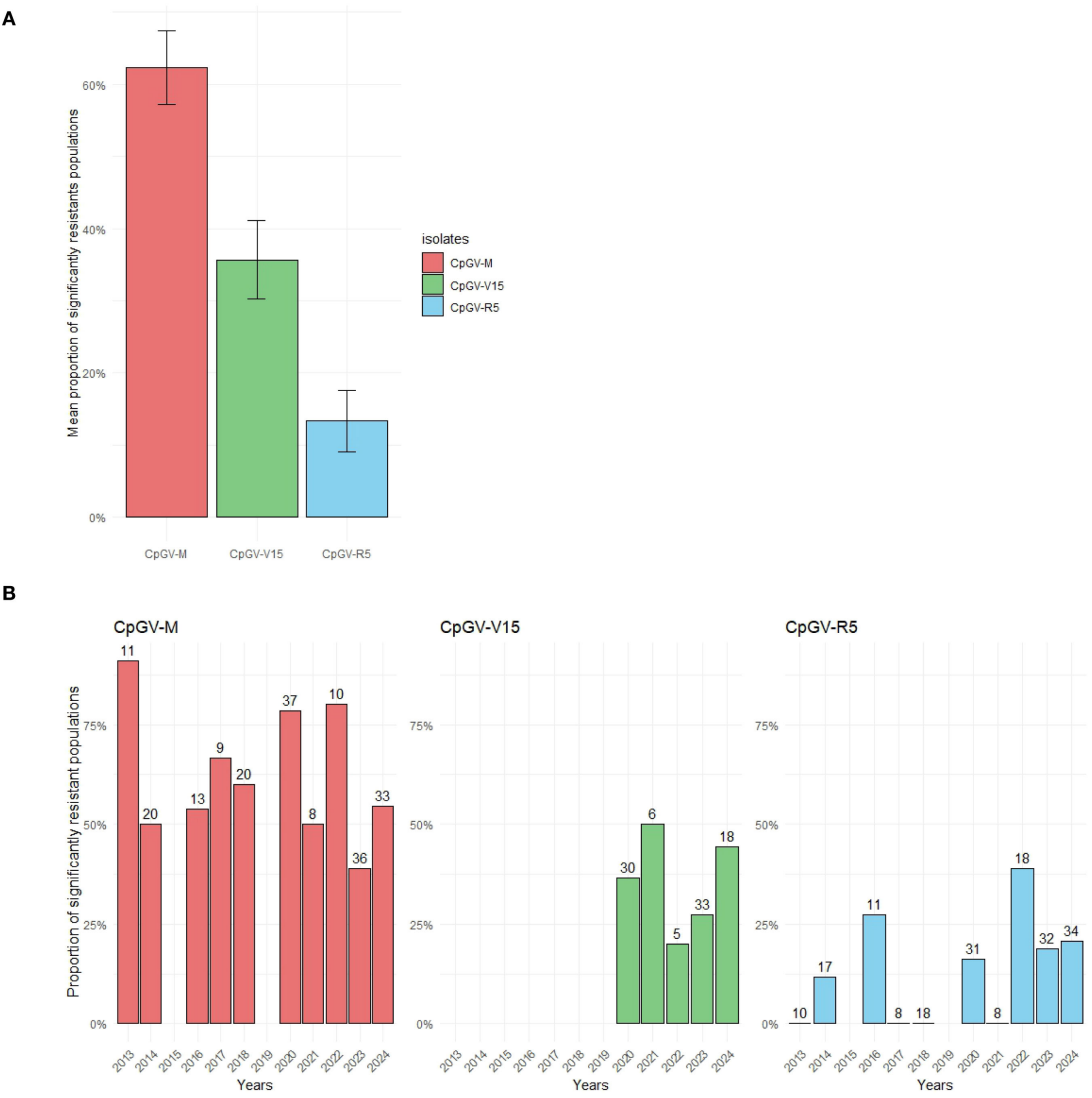
Evolution of resistance to the three viral isolates over time. Missing bar = no population tested this year. **(A)** Frequency of populations with a significant proportion of resistant individuals detected during the 12-year monitoring period. **(B)** Cumulative results from 12 years of monitoring.

These twelve years of monitoring *Cydia pomonella* resistance to CpGV have revealed resistant individuals to CpGV-M from the start, with an average frequency of 62.3% of the tested populations carrying a significant number of resistant individuals. Resistance monitoring for CpGV-V15 was only initiated in 2020. Even in this first year, 37% of the tested populations were found to carry a significant number of resistant individuals. In contrast, resistance to CpGV-R5 is less frequent, with an average of 13.3% of tested populations carrying resistant individuals across all years. These results indicate that, from 2020 onwards, we detected populations containing *Cydia pomonella* individuals resistant to all three viral isolates currently used in Europe (**Figure 2A**). No clear trend emerges regarding the evolution of the frequency of populations carrying resistant individuals for any of the viral isolates (**Figure 2B**).

**Figure 3:**
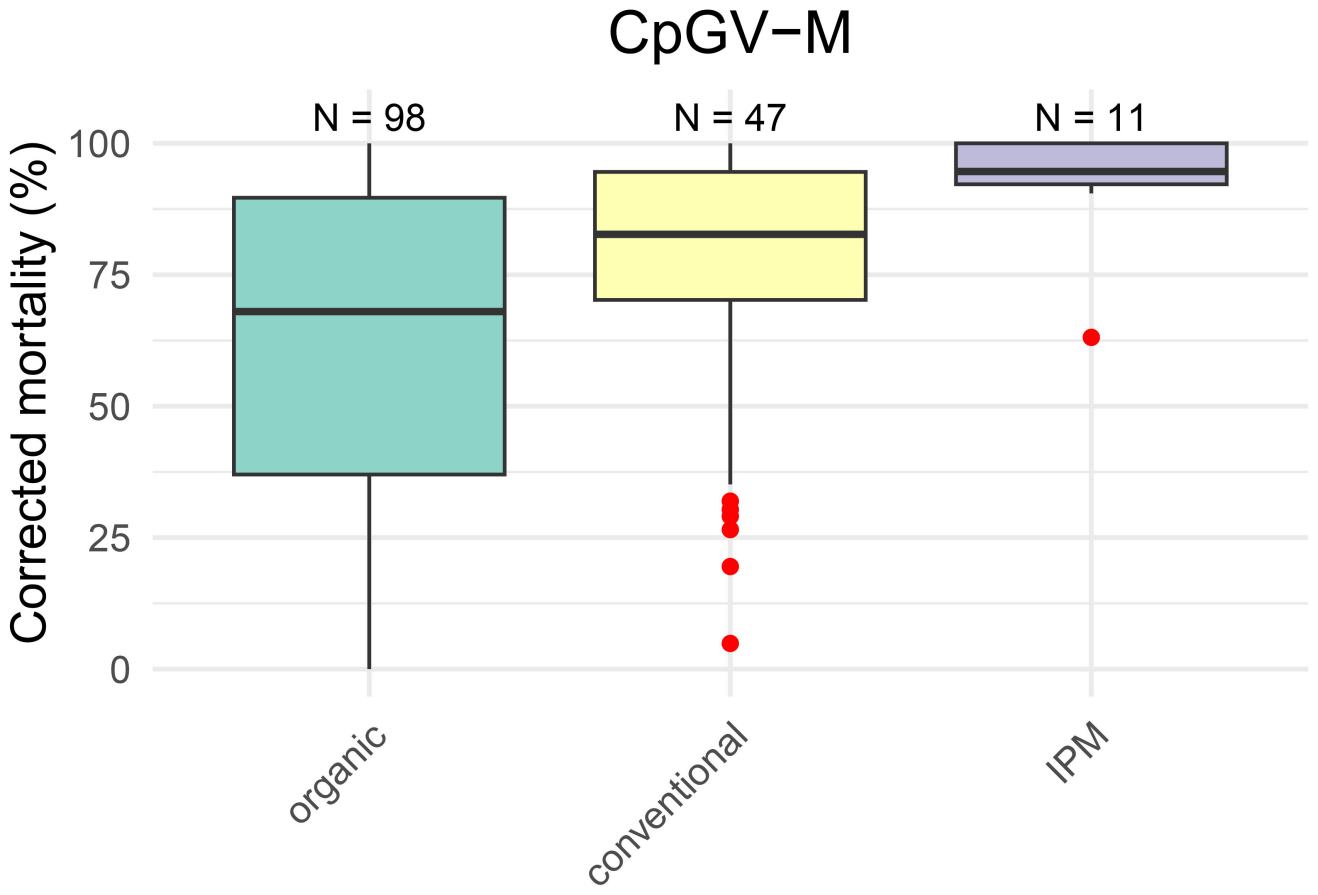
Effect of agricultural practices on the corrected mortality of wild populations of *C. pomonella* exposed to CpGV-M. Statistical test: ANOVA with a significance level of 0.05.

The number of treatments applied with CpGV-M, CpGV-V15, or CpGV-R5 during the year of sampling and the two preceding years on commercial apple orchards, where *C. pomonella* populations were collected, is not directly correlated with the sensitivity of these populations to each of these viruses (P = 0.163, sup mat 2). However, the type of cropping practice is a discriminating factor among the orchards. Indeed, populations sampled from orchards managed under organic farming practices exhibit, on average, lower corrected mortality rates when exposed to CpGV-M compared to those managed conventionally or according to integrated pest management principles (p<0.001; **Figure 3**).

**Figure 4:**
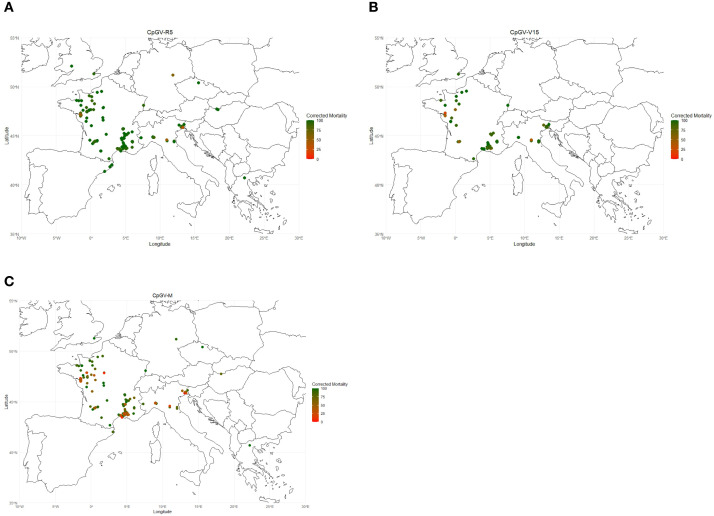
Geographical distribution of resistance to CpGV-R5 **(A)**; Cpgv-V15 **(B)** and CpGV-M **(C)** over 12 years of *C. pomonella* resistance monitoring. Each point represents a population.

Resistant individuals to CpGV-M were detected in multiple populations (around 60%) across the three major apple-producing regions in France, as well as in northern Italy (**Figure 4A**). Less frequent, individuals resistant to CpGV-V15 are mainly located in the Northwestern production basin in France and in the Modena region in Italy (**Figure 4B**). As for individuals resistant to CpGV-R5, they are also located in these two areas, but also in Germany, in the region where type II and III resistances have been described (Sauer et al., 2017), and in the Venice region in northeastern Italy (**Figure 4C**).

**Figure 5:**
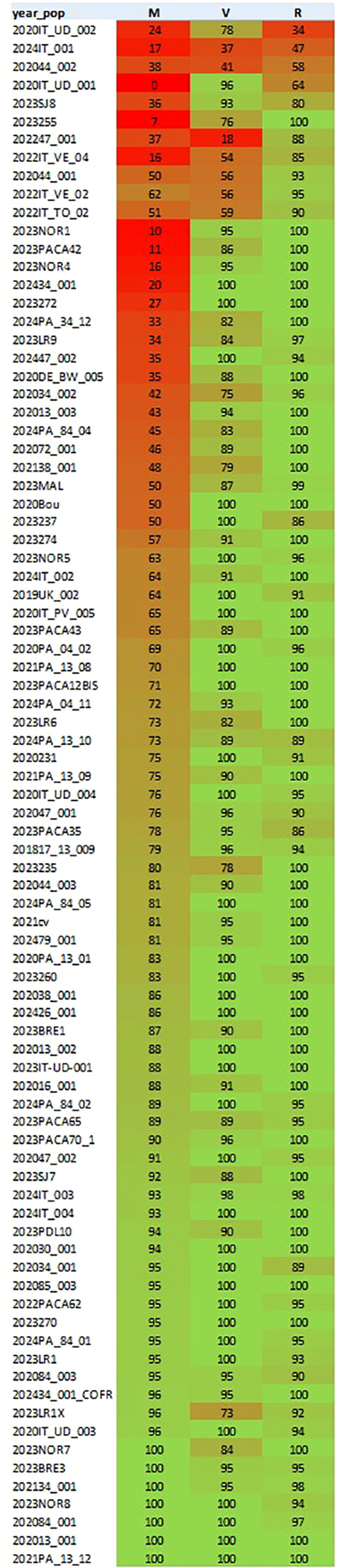
Table representing the monitoring data for which analyses could be performed on all three isolates. Each row represents a population, and the columns correspond to each isolate (M for CpGV-M; R for CpGV-R5 and V for CpGV-V15). The table is filled with Abbott-corrected mortalities at the discriminating dose, and the colors are chosen based on a gradient ranging from red (resistant) to green (susceptible).

Although the bioassay method does not allow us to determine whether a single individual carries resistance to multiple viral isolates, we observe a correlation between populations carrying individuals resistant to CpGV-R5 and those carrying individuals resistant to CpGV-M (Chi² = 12.8; p < 0.001). The same correlation is observed between populations carrying individuals resistant to CpGV-V15 and CpGV-M (Chi² = 15.1; p < 0.001). However, populations carrying individuals resistant to CpGV-R5 and CpGV-V15 are not significantly the same (Chi² = 2.4; p = 0.117) (**Figure 5**).

## Discussion

This resistance monitoring program for *Cydia pomonella* against various CpGV isolates marketed in Europe has revealed a high prevalence of populations containing individuals resistant to CpGV-M. This so-called Type I resistance has been known since 2006 and is already the focus of management programs that restrict the use of products based solely on CpGV-M in regions where this resistance is established. However, no decline in resistance prevalence has been observed over time.

This failure in resistance management can be attributed to two main factors:

(i) The absence of detectable fitness costs associated with this resistance (Siegwart et al., 2013; Undorf-Spahn et al., 2012), which slows the counter-selection of the resistance allele, and (ii) The new CpGV isolates used against resistant insects are, in fact, composed of mixtures from different genotypic groups—including Group A, to which CpGV-M belongs (Gueli Alletti et al., 2017). This inadvertently maintains selection pressure on Type I resistance.

These findings raise critical questions regarding the future of resistance management strategies. If we hope to establish an effective rotation strategy against Type I resistance, it is essential to develop and market CpGV products entirely free of Group A viruses.

In this study, we used the biotest method to monitor Type I resistance and to potentially detect resistance to CpGV-R5 and CpGV-V15 for the first time. This method was chosen due to the lack of prior knowledge on the genetic mechanisms behind any new resistance. However, the method is experimentally demanding, particularly due to challenges in rearing wild populations under laboratory conditions and breaking diapause. These constraints limit the number of individuals and populations that can be tested. We mitigated this by conducting observations over 12 years, thereby expanding the number of populations analyzed. Translating our results into field-level recommendations remains challenging. The frequency of resistant individuals is a key factor determining treatment failure risk. As a result, we only make resistance management recommendations when resistance is confirmed in the same orchard over several consecutive years. It is important to note that orchards included in this study were not randomly selected because analyses were only possible on samples containing at least 50 individuals. This implies that the plots for which we have results involve high levels of *C. pomonella* infestation which could be due to treatment failure. Therefore, the observed prevalence of approximately 60% for Type I resistance likely overestimates the actual picture.

As a result, we detected a significant number of orchards harboring individuals resistant to CpGV-R5 and CpGV-V15—an unprecedented finding. We have termed resistance to CpGV-M and CpGV-V15 as Type IV resistance, and resistance to CpGV-M, CpGV-V15, and CpGV-R5 as Type V resistance (Siegwart et al., 2020). At this stage, it is unclear whether these cases involve multiple resistance alleles that accumulated over successive treatments, or if it’s the consequence of cross-resistance where a single allele confers resistance to multiple CpGV isolates. A recent study (Gingueneau et al., 2025) suggests an intermediate scenario for type V resistance. This resistance seems to be the consequence of different linked mutations as exposure to any one of the three isolates selects for resistance to all three. But they have distinct inheritance patterns. CpGV-M resistance is sex-linked and dominant, whereas CpGV-R5 resistance appears to be sex-linked and mostly recessive. This supports the hypothesis that type V resistance results from the accumulation of two closely linked alleles on the Z chromosome. The management of this multiple resistance will thus be particularly complex.

Although less is known about Type IV resistance, our correlation analyses indicate a possible genetic link with Type I resistance. Surprisingly, we did not observe a strong correlation between resistance to CpGV-R5 and CpGV-V15, despite both isolates being at least 50% composed of Group E viruses (Gueli Alletti et al., 2017). This suggests that insects could potentially exhibit resistance to one isolate and susceptibility to the other, even within the same genotypic group. Therefore, the genetic mechanisms underlying Types IV and V resistance appear to be distinct.

These observations further support the idea that resistance-breaking ability may not solely depend on broad genotypic group classification (Jehle et al., 2017). Indeed, our results, together with those recently obtained by the German research team, indicate that sufficient genetic diversity can exist within a single genotypic group to generate important phenotypic differences. For example, several isolates belonging to genotypic group A, including CpGV-V03, are capable of overcoming Type I resistance despite their close relationship with CpGV-M (Jehle et al., 2026). Since Type I resistance is thought to rely on a simple genetic determinism involving a major gene, resistance-breaking in viral genomes may also depend on relatively limited genetic changes rather than on highly divergent genomes (Gebhardt et al., 2014).

This hypothesis is also consistent with the way new viral isolates are developed commercially. Most laboratories start from limited viral diversity and select variants capable of infecting resistant insects. As a result, resistance-breaking isolates often differ from their parental strains only by minor genetic modifications while still belonging to the same genotypic group.

In addition, conventional sequencing approaches may underestimate the complexity of these viral populations. The work of Gueli Alletti et al. (2017) demonstrated that recombination events between variants and even between genotypic groups can occur. However, current isolate characterization mainly relies on the quantification of group-specific SNPs, without mapping these SNPs along individual viral genomes. Consequently, it remains difficult to determine whether detected signatures correspond to the coexistence of distinct genotypic groups or to recombinant genomes containing fragments from multiple groups. Such recombination events could further contribute to the complexity of resistance-breaking mechanisms.

In our study, Type I resistance was detected in all regions surveyed. The lack of clear spatial structuring may reflect one of two scenarios: either a single resistance event spread across regions via long-distance dispersal despite the codling moth being generally sedentary or resistant individuals were already present across all regions before widespread CpGV use. The latter hypothesis is supported by another study showing the near-simultaneous emergence of Type I resistance in multiple European countries without any clear epidemiological connection (Schmitt et al., 2013). Our findings also align with this, as we observed that susceptible and resistant populations often coexist in close proximity, which seems inconsistent with a wave-like spread pattern.

We attempted to correlate resistance levels with the number of CpGV-based treatments, but surprisingly found no clear relationship. This is consistent with previous studies on resistance of *C. pomonella* to pyrethroids by the target mutation *kdr* (Franck et al., 2012) demonstrating that the number of treatments over a short timescale is insufficient to account for the resistance distribution. However, we did observe that Type I resistance was statistically more prevalent in organic orchards compared to those under Integrated Pest Management (IPM) or conventional practices. This is consistent with the fact that organic farmers apply CpGV-M more frequently than other growers. This supports the hypothesis that resistance is driven by long-term, uniform and repeated selection pressure in organic systems, where alternative pest control options are limited. This seems to suggest that the number of treatments in a single year is not a sufficiently robust indicator of selection pressure, but sustainable farming practices are indeed driving the evolution of pests such as the codling moth.

Interestingly, conventional plots showed more resistance than IPM-managed ones. This could be explained by the frequent use of CpGV in conventional farming due to its short pre-harvest interval, and the narrower range of pest control methods compared to IPM systems, which likely help prevent resistance buildup.

Looking ahead, viral cooperation strategies involving mixtures of mutually reinforcing CpGV strains should be carefully designed. These should avoid maintaining existing resistance in the field while promoting diverse infection pathways to delay the evolution of new resistance. The alternating use of different baculoviruses, such as nucleopolyhedroviruses, may offer promising solutions (Hinsberger et al., 2021; Wennmann et al., 2019).

## Conclusion

In conclusion, this long-term monitoring study shows that resistance of *Cydia pomonella* to CpGV isolates is now widespread in Europe and involves several resistance phenotypes beyond the well-known Type I resistance. The detection of Type IV and Type V resistances highlights the increasing complexity of resistance mechanisms and the limits of current management strategies based on resistance-breaking isolates.

Our results also suggest that viral efficacy cannot be explained solely by broad genotypic group classification. Minor genetic differences within the same group, as well as recombination events between variants, may strongly influence the ability of isolates to overcome resistance. This complexity is probably underestimated by current sequencing approaches.

These findings underline the need to rethink CpGV resistance management strategies. In particular, reducing continuous selection pressure and increasing the functional diversity of viral products will likely be essential to maintain the long-term efficacy of baculovirus-based control strategies against codling moth populations.

## Data Availability

The raw data supporting the conclusions of this article will be made available by the authors, without undue reservation.
