# Supplementary material for: Emergence of new resistances to Cydia pomonella Granulovirus: insights from 12 years of monitoring
Source: Front Physiol. 2026 Jul 8;17:1847124. doi: 10.3389/fphys.2026.1847124 (PMC13388200; doi:10.3389/fphys.2026.1847124)
Supplement: Supplementary file 1 [file Table1.docx]

Sup mat 1 : list of tested populations of *Cydia pomonella*

| pop_code | year | municipality | postal code | country | tested isolates |
| --- | --- | --- | --- | --- | --- |
| 2013_13550_1 | 2013 | les_paluds_de_noves | 13550 | France | M, R5 |
| 2013_13940_2 | 2013 | mollegues | 13940 | France | M, R5 |
| 2013_13940_3 | 2013 | molleges | 13940 | France | M |
| 2013_13940_4 | 2013 | molleges | 13940 | France | M, R5 |
| 2013_13940_5 | 2013 | molleges | 13940 | France | M, R5 |
| 2013_26240_6 | 2013 | beausemblant | 26240 | France | M, R5 |
| 2013_47500_7 | 2013 | fumel | 47500 | France | M, R5 |
| 2013_84300_8 | 2013 | cavaillon | 84300 | France | M, R5 |
| 2013_13670_9 | 2013 | saint_andiol | 13670 | France | M, R5 |
| 2013_26270_10 | 2013 | loriol_sur_drome | 26270 | France | M, R5 |
| 2013_38570_11 | 2013 | le_cheylas | 38570 | France | M, R5 |
| 2014_69510_11 | 2014 | messimy | 69510 | France | M, R5 |
| 2014_69510_12 | 2014 | messimy | 69510 | France | M, R5 |
| 2014_81500_13 | 2014 | ambres | 81500 | France | M, R5 |
| 2014_84460_14 | 2014 | cheval_blanc | 84460 | France | M, R5 |
| 2014_50448_15 | 2014 | saint_aubin_ de_terregatte | 50448 | France | M, R5 |
| 2014_14190_16 | 2014 | cinq_autel_valambray | 14190 | France | M, R5 |
| 2014_13670_17 | 2014 | saint_andiol | 13670 | France | M, R5 |
| 2014_53200_18 | 2014 | longuefuye | 53200 | France | M, R5 |
| 2014_47500_19 | 2014 | saint_vite | 47500 | France | M |
| 2014_30159_20 | 2014 | manduel | 30159 | France | M |
| 2014_13690_21 | 2014 | graveson | 13690 | France | M |
| 2014_36230_22 | 2014 | neuvy_saint_sepulchre | 36230 | France | M, R5 |
| 2014_37530_23 | 2014 | l_aubiniere | 37530 | France | M |
| 2014_36128_24 | 2014 | la_fleuranderie | 36128 | France | M, R5 |
| 2014_84460_25 | 2014 | cheval_blanc | 84460 | France | M, R5 |
| 2014_45160_26 | 2014 | saint_hilaire_saint_mesmin | 45160 | France | M |
| 2014_13940_27 | 2014 | molleges | 13940 | France | M, R5 |
| 2015_47500_29 | 2015 | saint_vite | 47500 | France | M, R5 |
| 2015_13370_30 | 2015 | mallemort | 13370 | France | M, R5 |
| 2015_84300_31 | 2015 | cavaillon | 84300 | France | M, R5 |
| 2016_47320_32 | 2016 | laffite_sur_lot | 47320 | France | M |
| 2016_18110_33 | 2016 | saint_martin_d_auxigny | 18110 | France | M,R5 |
| 2016_45160_34 | 2016 | saint_hilaire_saint_mesmin | 45160 | France | M, R5 |
| 2016_45160_35 | 2016 | saint_hilaire_saint_mesmin | 45160 | France | M, R5 |
| 2016_30300_36 | 2016 | jonquiere_saint_vincent | 30300 | France | M, R5 |
| 2016_30300_37 | 2016 | jonquiere_saint_vincent | 30300 | France | M |
| 2016_13670_38 | 2016 | saint_andiol | 13670 | France | M, R5 |
| 2016_49130_39 | 2016 | les_ponts_de_ce | 49130 | France | M, R5 |
| 2016_44470_40 | 2016 | thouare_sur_loire | 44470 | France | M, R5 |
| 2016_44470_41 | 2016 | carquefou | 44470 | France | M, R5 |
| 2016_13570_42 | 2016 | barbentane | 13570 | France | M, R5 |
| 2016_13590_43 | 2016 | graveson | 13590 | France | M, R5 |
| 2016_30129_44 | 2016 | redessan | 30129 | France | M, R5 |
| 2017_13660_45 | 2017 | orgon | 13660 | France | M, R5 |
| 2017_13370_46 | 2017 | mallemort | 13370 | France | M, R5 |
| 2017_13670_47 | 2017 | saint_andiol | 13670 | France | M, R5 |
| 2017_13670_48 | 2017 | saint_andiol | 13670 | France | M, R5 |
| 2017_84300_49 | 2017 | cavaillon | 84300 | France | M, R5 |
| 2017_26300_50 | 2017 | chateneuf_sur_isere | 26300 | France | M, R5 |
| 2017_26270_51 | 2017 | loriol_sur_drome | 26270 | France | M, R5 |
| 2017_69510_52 | 2017 | soucieu_en_jarrest | 69510 | France | M, R5 |
| 2018_13570_53 | 2018 | barbentane | 13570 | France | M |
| 2018_84100_54 | 2018 | orange | 84100 | France | M, R5 |
| 2018_84100_55 | 2018 | orange | 84100 | France | M, R5 |
| 2018_49150_56 | 2018 | chevire_le_rouge | 49150 | France | M, R5 |
| 2018_49130_57 | 2018 | les_ponts_de_ce | 49130 | France | M, R5 |
| 2018_47260_58 | 2018 | granges_sur_lot | 47260 | France | M, R5 |
| 2018_30300_59 | 2018 | jonquiere_saint_vincent | 30300 | France | M, R5 |
| 2018_44140_60 | 2018 | la_planche | 44140 | France | M, R5 |
| 2018_47110_61 | 2018 | le_temple_sur_lot | 47110 | France | M, R5 |
| 2018_44140_62 | 2018 | la_planche | 44140 | France | M, R5 |
| 2018_30300_63 | 2018 | beaucaire | 30300 | France | M, R5 |
| 2018_26320_64 | 2018 | saint_marcel_les_valences | 26320 | France | M, R5 |
| 2018_69510_65 | 2018 | soucieu_en_jarrest | 69510 | France | M, R5 |
| 2018_30300_66 | 2018 | jonquiere_saint_vincent | 30300 | France | M, R5 |
| 2018_44410_67 | 2018 | carquefou | 44410 | France | M, R5 |
| 2018_37370_68 | 2018 | saint_aubin_le_depeint | 37370 | France | M, R5 |
| 2018_53200_69 | 2018 | longuefuye | 53200 | France | M, R5 |
| 2018_37800_70 | 2018 | saint_epain | 37800 | France | M, R5 |
| 2018_13370_71 | 2018 | mallemort | 13370 | France | M, R5, V15 |
| 2018_49430_72 | 2018 | montigne_les_prairies | 49430 | France | R5 |
| 2019_ME196BJ_73 | 2019 | east_malling | ME196BJ | UK | M, R5, V15 |
| 2020_13570_74 | 2020 | barbentane | 13570 | France | M, R5, V15 |
| 2020_13570_75 | 2020 | barbentane | 13570 | France | M, R5, V15 |
| 2020_13570_76 | 2020 | barbentane | 13570 | France | M, R5, V15 |
| 2020_44410_77 | 2020 | carquefou | 44410 | France | M, R5, V15 |
| 2020_84100_78 | 2020 | orange | 84100 | France | M, R5, V15 |
| 2020_84100_79 | 2020 | orange | 84100 | France | M |
| 2020_13670_80 | 2020 | saint_andiol | 13670 | France | M, R5 |
| 2020_13940_81 | 2020 | molleges | 13940 | France | M, R5, V15 |
| 2020_44140_82 | 2020 | la_planche | 44140 | France | M, R5, V15 |
| 2020_79206_83 | 2020 | breisach | 79206 | Germany | M, R5, V15 |
| 2020_84300_84 | 2020 | cavaillon | 84300 | France | M, R5, V15 |
| 2020_44140_85 | 2020 | la_planche | 44140 | France | M, R5, V15 |
| 2020_33046_86 | 2020 | pulfero | 33046 | Italy | M, R5, V15 |
| 2020_33050_87 | 2020 | bagnaria_arsa | 33050 | Italy | M, R5, V15 |
| 2020_27050_88 | 2020 | bagnaria | 27050 | Italy | M, R5, V15 |
| 2020_33061_89 | 2020 | rivignano | 33061 | Italy | M, R5, V15 |
| 2020_72800_90 | 2020 | thoree_les_pins | 72800 | France | M, R5, V15 |
| 2020_15050_91 | 2020 | brignano_frascata | 15050 | Italy | M |
| 2020_47110_92 | 2020 | le_temple_sur_lot | 47110 | France | M, R5, V15 |
| 2020_84100_93 | 2020 | orange | 84100 | France | M, R5, V15 |
| 2020_47440_94 | 2020 | casseneuil | 47440 | France | M, R5, V15 |
| 2020_33050_95 | 2020 | mortegliano_udime | 33050 | Italy | M |
| 2020_33099_96 | 2020 | vivaro | 33099 | Italy | M, R5, V15 |
| 2020_15052_97 | 2020 | casalnoceto | 15052 | Italy | M, R5 |
| 2020_15029_98 | 2020 | monleale | 15029 | Italy | M |
| 2020_34590_99 | 2020 | marsillargues | 34590 | France | M, R5, V15 |
| 2020_34590_100 | 2020 | marsillargues | 34590 | France | M, R5, V15 |
| 2020_4250_101 | 2020 | la_motte_du_caire | 4250 | France | M, R5, V15 |
| 2020_84300_102 | 2020 | cavaillon | 84300 | France | M, R5, V15 |
| 2020_85240_103 | 2020 | saint_hilaire_des_loges | 85240 | France | M, R5, V15 |
| 2020_85240_104 | 2020 | saint_hilaire_des_loges | 85240 | France | M |
| 2020_30300_105 | 2020 | jonquiere_saint_vincent | 30300 | France | M, R5, V15 |
| 2020_16700_106 | 2020 | la_faye | 16700 | France | M, R5, V15 |
| 2020_38210_107 | 2020 | Tullin | 38210 | France | M, R5, V15 |
| 2021_34400_108 | 2021 | saint_just | 34400 | France | M, R5, V15 |
| 2021_38160_109 | 2021 | saint_hilaire_du_rosier | 38160 | France | M, R5, V15 |
| 2021_13370_110 | 2021 | mallemort | 13370 | France | M, R5, V15 |
| 2021_13560_111 | 2021 | senas | 13560 | France | M, R5, V15 |
| 2021_13560_112 | 2021 | senas | 13560 | France | M, R5 |
| 2021_84300_113 | 2021 | cavaillon | 84300 | France | M, R5 |
| 2021_4210_114 | 2021 | manosque_valensole | 4210 | France | M, R5, V15 |
| 2021_84460_115 | 2021 | cheval_blanc | 84460 | France | M, R5, V15 |
| 2022_47260_116 | 2022 | granges_sur_lot | 47260 | France | M, R5, V15 |
| 2022_47110_117 | 2022 | le_temple_sur_lot | 47110 | France | M, R5 |
| 2022_84460_118 | 2022 | cheval_blanc | 84460 | France | M, R5 |
| 2022_2921_119 | 2022 | koma_rom | 2921 | Hungary | M, R5 |
| 2022_27050_120 | 2022 | bagnaria | 27050 | Italy | M, R5, V15 |
| 2022_15050_121 | 2022 | montemarzino | 15050 | Italy | M, R5 |
| 2022_33050_122 | 2022 | mortegliano_udime | 33050 | Italy | M, R5, V15 |
| 2022_33050_123 | 2022 | ariis_di_rivignano_udime | 33050 | Italy | M, R5 |
| 2022_33050_124 | 2022 | pavla_di_udime | 33050 | Italy | M, R5, V15 |
| 2022_84840_125 | 2022 | la_motte_du_rhone | 84840 | France | M, R5, V15 |
| 2022_44690_126 | 2022 | maisdon_sur_sevre | 44690 | France | R5 |
| 2022_47440_127 | 2022 | casseneuil | 47440 | France | R5 |
| 2022_49150_128 | 2022 | montigne_les_prairies | 49150 | France | R5 |
| 2022_17455_129 | 2022 | caldes_de_malavella | 17455 | Spain | R5 |
| 2022_2836_130 | 2022 | bas | 2836 | Hungary | R5 |
| 2022_15050_131 | 2022 | brignano_frascata | 15050 | Italy | R5 |
| 2022_33046_132 | 2022 | pulfero_udime | 33046 | Italy | R5 |
| 2022__133 | 2022 | upper_lyde_herefordshire |  | UK | R5 |
| 2023_13560_134 | 2023 | senas | 13560 | France | M, R5, V15 |
| 2023_13560_135 | 2023 | senas | 13560 | France | M, R5, V15 |
| 2023_84250_136 | 2023 | le_thor | 84250 | France | M, R5, V15 |
| 2023_84800_137 | 2023 | l_isle_sur_la_sorgue | 84800 | France | M, R5, V15 |
| 2023_84460_138 | 2023 | cheval_blanc | 84460 | France | M, R5, V15 |
| 2023_84460_139 | 2023 | cheval_blanc | 84460 | France | M, R5, V15 |
| 2023_13750_140 | 2023 | plan_d_orgon | 13750 | France | M, R5, V15 |
| 2023_35730_141 | 2023 | pleurtuit | 35730 | France | M, R5, V15 |
| 2023_35120_142 | 2023 | quercout | 35120 | France | M, R5 |
| 2023_35113_143 | 2023 | domagne | 35113 | France | M, R5, V15 |
| 2023_84460_144 | 2023 | cheval_blanc | 84460 | France | M, V15 |
| 2023_84460_145 | 2023 | cheval_blanc | 84460 | France | M, V15 |
| 2023_33050_146 | 2023 | mortegliano_udime | 33050 | Italy | M, R5, V15 |
| 2023_34130_147 | 2023 | lansargues | 34130 | France | M, R5, V15 |
| 2023_34130_148 | 2023 | lansargues | 34130 | France | M, R5, V15 |
| 2023_34590_149 | 2023 | marsillargues | 34590 | France | M, V15 |
| 2023_66130_150 | 2023 | corbere | 66130 | France | M, R5, V15 |
| 2023_34730_151 | 2023 | prades_le_lez | 34730 | France | M, R5, V15 |
| 2023_13200_152 | 2023 | arles | 13200 | France | M, R5, V15 |
| 2023_61500_153 | 2023 | sees | 61500 | France | M, R5, V15 |
| 2023_61260_154 | 2023 | l_hermitiere | 61260 | France | M, R5, V15 |
| 2023_14140_155 | 2023 | heurtevent | 14140 | France | M, R5, V15 |
| 2023_76_156 | 2023 | bremontier_merval | 76 | France | M, R5, V15 |
| 2023_76840_157 | 2023 | quevillons | 76840 | France | M, R5, V15 |
| 2023_13_158 | 2023 | tarascon | 13 | France | M, R5, V15 |
| 2023_13560_159 | 2023 | senas | 13560 | France | M, R5, V15 |
| 2023_13300_160 | 2023 | salon | 13300 | France | M, R5, V15 |
| 2023_13350_161 | 2023 | charleval | 13350 | France | M, R5, V15 |
| 2023_84300_162 | 2023 | cavaillon | 84300 | France | M, R5, V15 |
| 2023_5110_163 | 2023 | curbans | 5110 | France | M, R5, V15 |
| 2023_44120_164 | 2023 | vertou | 44120 | France | M, R5, V15 |
| 2023_44370_165 | 2023 | varades | 44370 | France | M |
| 2023_26270_166 | 2023 | loriol_sur_drome | 26270 | France | M, R5 |
| 2023_13670_167 | 2023 | saint_andiol | 13670 | France | M, R5, V15 |
| 2023_13670_168 | 2023 | saint_andiol | 13670 | France | M, R5, V15 |
| 2024_26260_169 | 2024 | arthemonay | 26260 | France | M, R5, V15 |
| 2024_34590_170 | 2024 | marsillargues | 34590 | France | M, R5, V15 |
| 2024_79300_171 | 2024 | bressuire | 79300 | France | M, R5, V15 |
| 2024_5110_172 | 2024 | curbans | 5110 | France | M, R5, V15 |
| 2024_13670_173 | 2024 | saint_andiol | 13670 | France | M, R5, V15 |
| 2024_34590_174 | 2024 | marsillargues | 34590 | France | M, R5, V15 |
| 2024_84250_175 | 2024 | le_thor | 84250 | France | M, R5, V15 |
| 2024_84300_176 | 2024 | les_vignieres | 84300 | France | M, R5, V15 |
| 2024_84300_177 | 2024 | cavaillon | 84300 | France | M, R5, V15 |
| 2024_84250_178 | 2024 | le_thor | 84250 | France | M, R5, V15 |
| 2024_13570_179 | 2024 | barbentane | 13570 | France | M, R5 |
| 2024_30490_180 | 2024 | montfrin | 30490 | France | M, R5 |
| 2024_34590_181 | 2024 | marsillargues | 34590 | France | M, R5, V15 |
| 2024_44120_182 | 2024 | vertou | 44120 | France | M, R5 |
| 2024_44140_183 | 2024 | la_planche | 44140 | France | M, R5 |
| 2024_44140_184 | 2024 | remouille | 44140 | France | M, R5 |
| 2024_33190_185 | 2024 | pondaurat | 33190 | France | M, R5 |
| 2024_47110_186 | 2024 | le_temple_sur_lot | 47110 | France | M, R5, V15 |
| 2024_49130_187 | 2024 | les_ponts_de_ce | 49130 | France | M, R5 |
| 2024_49480_188 | 2024 | saint_sylvain_d_anjou | 49480 | France | M, R5 |
| 2024_27050_189 | 2024 | bagnaria | 27050 | Italy | M, R5 |
| 2024_10060_190 | 2024 | via_cascinasada_bibiana | 10060 | Italy | M, R5 |
| 2024_33050_191 | 2024 | mortegliano_udime | 33050 | Italy | M, R5 |
| 2024_50752_192 | 2024 | ostromef | 50752 | Czech Republic | M, R5 |
| 2024_6667_193 | 2024 | goseck | 6667 | Germany | M, R5 |
| 2024_17131_194 | 2024 | parlava | 17131 | Spain | M, R5 |
| 2024_58500_195 | 2024 | arseni | 58500 | Greece | M, R5 |
| 2024_41123_196 | 2024 | modena | 41123 | Italy | M, R5, V15 |
| 2024__197 | 2024 | bagnacavallo_lo_villanova |  | Italy | M, R5, V15 |
| 2024_41058_198 | 2024 | vignola | 41058 | Italy | M, R5, V15 |
| 2024__199 | 2024 | russi |  | Italy | M, R5, V15 |
| 2024_8970_200 | 2024 | sant_joan_despi | 8970 | Spain | R5 |
