# Supplementary figures and images for: Emergence of new resistances to Cydia pomonella Granulovirus: insights from 12 years of monitoring
Source: Front Physiol. 2026 Jul 8;17:1847124. doi: 10.3389/fphys.2026.1847124 (PMC13388200; doi:10.3389/fphys.2026.1847124)

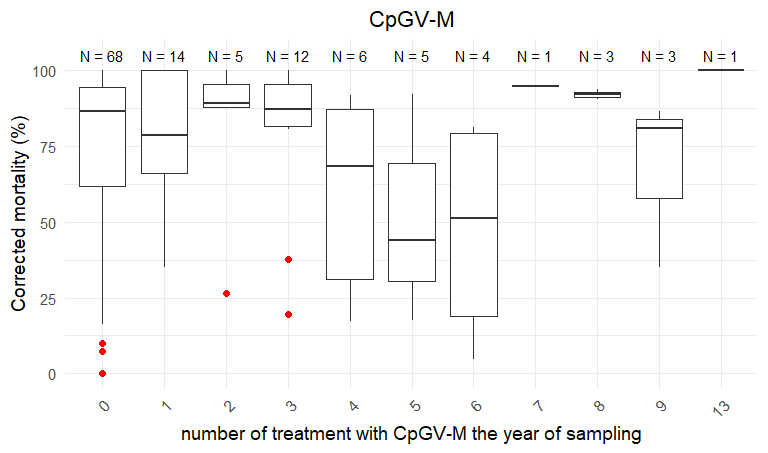

Supplement: Supplementary file 2 [file Image1.png]
